# Numerical Simulation on Zonal Disintegration in Deep Surrounding Rock Mass

**DOI:** 10.1155/2014/379326

**Published:** 2014-01-30

**Authors:** Xuguang Chen, Yuan Wang, Yu Mei, Xin Zhang

**Affiliations:** ^1^Institute of Tunnel and Urban Railway Engineering, Key Laboratory of Ministry of Education for Geomechanics and Embankment Engineering, Hohai University, Nanjing 210098, China; ^2^State Key Laboratory for GeoMechanics and Deep Underground Engineering, China University of Mining & Technology, Xuzhou 221000, China

## Abstract

Zonal disintegration have been discovered in many underground tunnels with the increasing of embedded depth. The formation mechanism of such phenomenon is difficult to explain under the framework of traditional rock mechanics, and the fractured shape and forming conditions are unclear. The numerical simulation was carried out to research the generating condition and forming process of zonal disintegration. Via comparing the results with the geomechanical model test, the zonal disintegration phenomenon was confirmed and its mechanism is revealed. It is found to be the result of circular fracture which develops within surrounding rock mass under the high geostress. The fractured shape of zonal disintegration was determined, and the radii of the fractured zones were found to fulfill the relationship of geometric progression. The numerical results were in accordance with the model test findings. The mechanism of the zonal disintegration was revealed by theoretical analysis based on fracture mechanics. The fractured zones are reportedly circular and concentric to the cavern. Each fracture zone ruptured at the elastic-plastic boundary of the surrounding rocks and then coalesced into the circular form. The geometric progression ratio was found to be related to the mechanical parameters and the ground stress of the surrounding rocks.

## 1. Introduction

**Figure 1:**
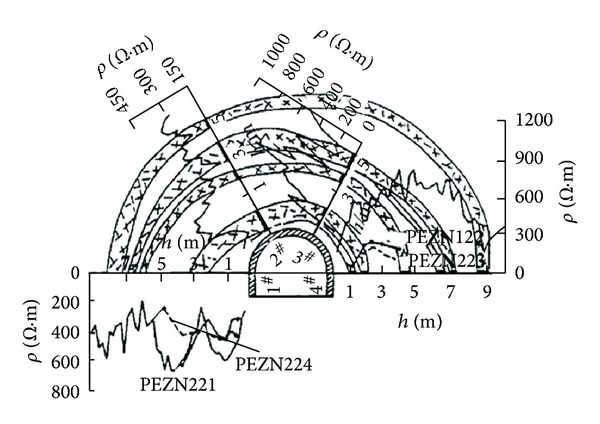
Zonal disintegration of Taimyrskii mine [4].

**Figure 2:**
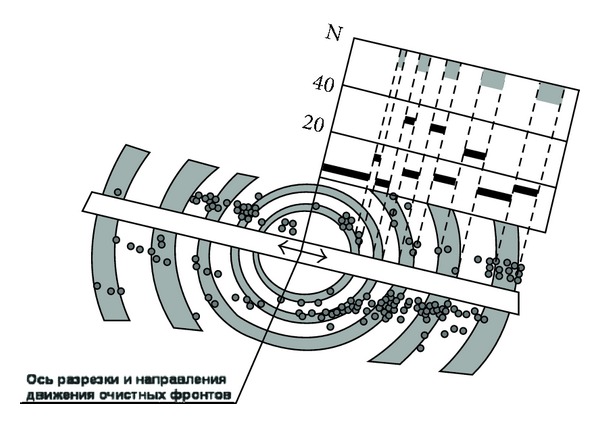
Relationship between the damage of support and the distribution of zonal disintegration [4].

Recently, many countries have begun to focus on deep resource exploitation. With an increase in embedded depth, the zonal disintegration phenomenon occurs during tunnel excavation. Zonal disintegration refers to “alternating regions of fractured and relatively intact rock masses appearing around or in front of the working stope during the excavation of tunnels in the deep rock mass” [1]. It is a special geological phenomenon that is different from what is observed for shallow embedded tunnels and presents a great hazard to the stability of deep surrounding rocks [2]. Adams and Jager (1980) first observed this phenomenon using a borehole periscope at an embedded depth of 2000–3000 m in the Witwatersrand gold mine, South Africa [3]. Shemyakin et al. [4–6] explored zonal disintegration by using a resistivity meter in the Taimyrskii deep mine, Russia (Figures 1 and 2). Zonal disintegration poses a great danger during the excavation of deep tunnels [7].

It is a character of the deep rock mass and has recently been a subject of focus. Many specialists have implemented several types of methods to explain the phenomenon. Sellers and Klerck [8] believed that discontinuous surfaces could be one of the origins of zonal disintegration. Malan and Spottiswoode [9] analyzed the relationship between shock bumps and zonal disintegration. Zhou et al. [10] studied the dynamic excavation of deep tunnels and determined the forming time of fractured zones. Gu et al. [11] carried out compressive tests on cylindrical specimens and believed axial stress to be the important cause of zonal disintegration. Some specialists have used nonequilibrium thermodynamics (Metlov L S, 2002), Hamilton time domain variation (LI SC, 2009), or a non-Euclidean model (GUZEV M A, 2001) to study the forming mechanism.

However, the mechanical behaviour of deep rock mass is nonlinear, very complex, and clearly different from the engineering response observed in shallow embedded tunnel engineering. The zonal structure of fracturing discovered does not fit within the framework of the conventional theoretical models. Indeed, there is no convincing explanation, but some arguments on the forming condition and failure mode have been made. For example, Oparin and Kurlenya [12] believe fractured zone are circular and concentric to the tunnel periphery and developed a formula for calculating the radii of fracture zones. On contrary, Borzykh [13] believes that fractured zones are a group of plastic slippage lines that form after deep rock mass yields plastically. In contrast to the results obtained in the above-mentioned studies, Tang and Zhang's [14] numerical simulation results show that the fractured zones appear more like spirals. Therefore, it is necessary to research the forming process and fracture pattern and thereby determine the underlying mechanism.

Simulation on the excavation process of tunnel in laboratory, using numerical method, is the most effective method. Based on this, the forming process and phenomena of zonal disintegration can be simulated and the mechanism can be revealed accordingly.

## 2. Materials and Methods

### 2.1. Introduction to the Model Test and Numerical Simulation

The two methods of physical and numerical simulation have their own merit and are complementary to each other. During the numerical simulation, virtual model is established in computer to research the actual engineering. It can be used to simulate the anisotropism, anisotropic and discontinuity characters of the medium, and the complex boundary condition in engineering.

Compared to numerical simulation, model test can reflect failure process in rock mass visually and truly. The analogical model test is an effective reduced-scale method for researching special engineering based on similarity theory. The model is constructed in a manner similar to that in which engineering prototypes and thus the deformation laws can be monitored using precision devices. The data from the model can be converted to that of an engineering prototype using similarity theory to reveal the stress distribution. Hence, real-world problems can be solved using this methodology.

The different advantages can be used and complementary to each other and thus the mechanism of the zonal disintegration can be revealed.

### 2.2. Model Test on Zonal Disintegration

The similarity theory required that the following similarity criteria must be satisfied in mechanical modeling (Fumagalli [15, 16]):

(1) $$\begin{array}{c} C_{\sigma }=C_{\gamma }C_{L},\\ C_{\delta }=C_{\varepsilon }C_{L},\\ C_{\sigma }=C_{\varepsilon }C_{E},\\ C_{\varepsilon }=C_{f}=C_{\phi }=C_{\mu }=1, \end{array}$$

where *C*_*L*_, *C*_*σ*_, *C*_*ε*_, *C*_*γ*_, *C*_*E*_, *C*_*μ*_, *C*_*ρ*_, *C*_*f*_, *C*_*c*_, and *C*_*φ*_ represent the similarity constants for geometry, stress, strain, volume weight, deformation modulus, Poisson's ratio, density, friction coefficient, and cohesion and friction angle, respectively.

To carry out the analogical model test, a suitable analogical material is required. The material determines whether the model test can reflect the mechanical response of the engineering prototype. Barite powder, iron powder, and quartz sand are used for the aggregate, and an alcohol solution of rosin is used as the mucilage glue. Through hundreds of sets of proportioning tests, a material referred to as Barites-Iron-Sand cementation analogical material was developed. The proportion of the aggregates and the concentration of the alcohol solution of rosin determine the mechanical behaviour of the material. The proportion of the aggregates and the concentration of the alcohol solution of rosin decide the mechanical behaviour of the material.

The similarity ratio of volume weight for the similar material is set to 1 : 1, while the similarity ratio of geostress is 1 : 25. Thus, the mechanical parameters of the mudstone and the corresponding similar material are shown in Table 1.

Construction of the model: the model size is limited by the reasonable size of the steel frame and 30 were taken as the optimal similarity coefficient *C*_*L*_ in the current study. The simulation range of the prototype was 11 m. According to the geometry similarity scale, the dimension of the model was determined to be 0.45 m and the model tunnel was 160 mm.

**Figure 3:**
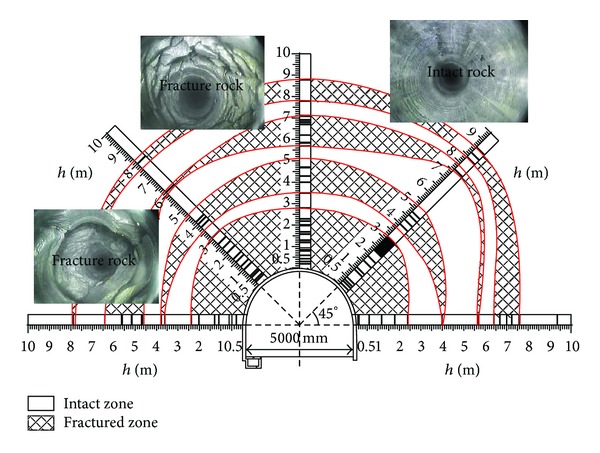
Zonal disintegration of Dingji coal mine of Huainan mine area.

The model was delaminated. The similar material was placed inside the steel drum in layers and then tamped to create the model. The steel drum was 400 mm high, and the wall was 10 mm thick and had an inner diameter of 450 mm. Each layer was 100 mm high, and thus four layers were needed to complete the model. Then, a circular cavern cylinder was preset inside the steel drum to make the tunnel. The cavern diameter was 160 mm and the cavern axis coincided with the central axes of the steel drum. Then the similar material was put inside the steel drum and compressed uniformly to build the model. The measuring components were set up when the material reached the desired height, to monitor the displacement in the surrounding rocks (Figure 3).

**Figure 4:**
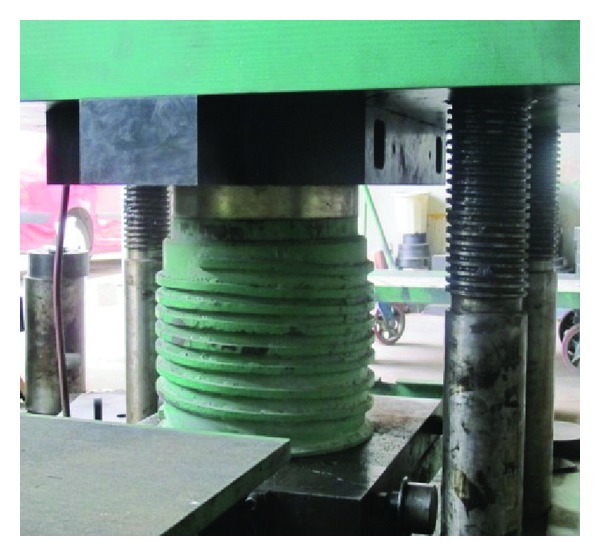
Loading procedure of model test.

The model was split and the cracks distribution inside the model was then determined (Figure 4). Circular cracks propagated and coalesced to form a circular pattern. Three to four fracture zones were observed to surround the cavern, in accordance with the fractured shape obtained from the field monitoring. Thus, the occurrence of zonal disintegration was confirmed in the model.

**Figure 5:**
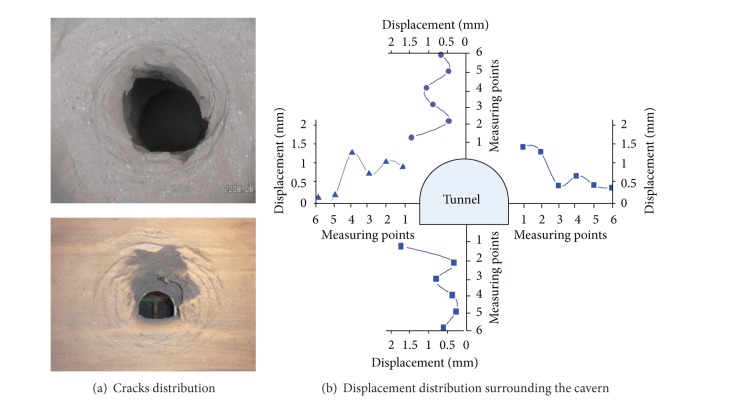
Results of the model test.

As Figure 5 shows, there appears about 3-4 fractured around the tunnel, which is the zonal disintegration. And during the zonal disintegration, it is found that the deformation of the rock mass shows a waved shape. The details could be found in the paper [17, 18].

### 2.3. Introduction to XFEM (Extended Finite Element Method)

In recent 5 years, many specialists have tried all kinds of numerical methods to simulate the zonal disintegration. Tang and Zhang [14] adapted RFPA and Qian et al. [19] and Wang [20] adapted strain soften model. However, the results are spiral, which is not consistent with the definition of zonal disintegration. So it is needed to find a new numerical method to research it.

The elements are needed to subdivide continuously during the crack propagation when using FEM. So it is not proper to the fracture problem in rock engineering. In 1999, Belytschko and Black [21] developed the XFEM. It is a newly developed means to simulate the fracture propagation for the rock materials. It can be considered as the extension of regular FEM. It has both the advantages of regular FEM and the special character of itself:

1. the fracture is not need to be considered. The element can be subdivided in any part, including the fracture;
2. the grid is not need to subdivide again, which improves the efficiency of computing highly;
3. the shape function can be changed according to the problem in study, which is very flexible.

**Figure 6:**
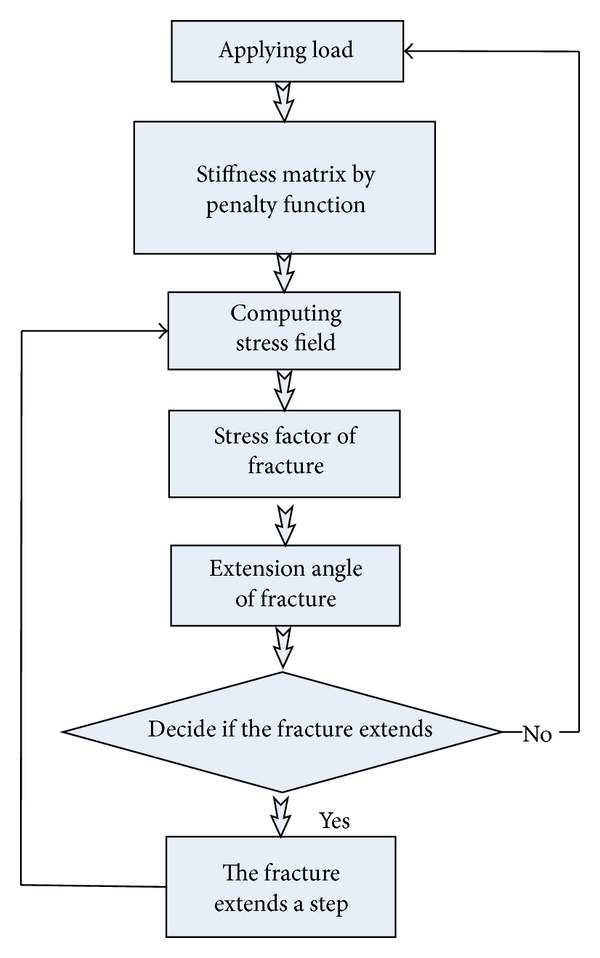
Numerical procedure of the extended finite element method (XFEM).

Many specialists have paid attention to XFEM and applied it to the rock fracture analysis. XFEM is adapted in this study with the criteria proposed to simulate the zonal disintegration. The procedure can be shown (Figure 6).

### 2.4. Simulations on the Crack Development

**Figure 7:**
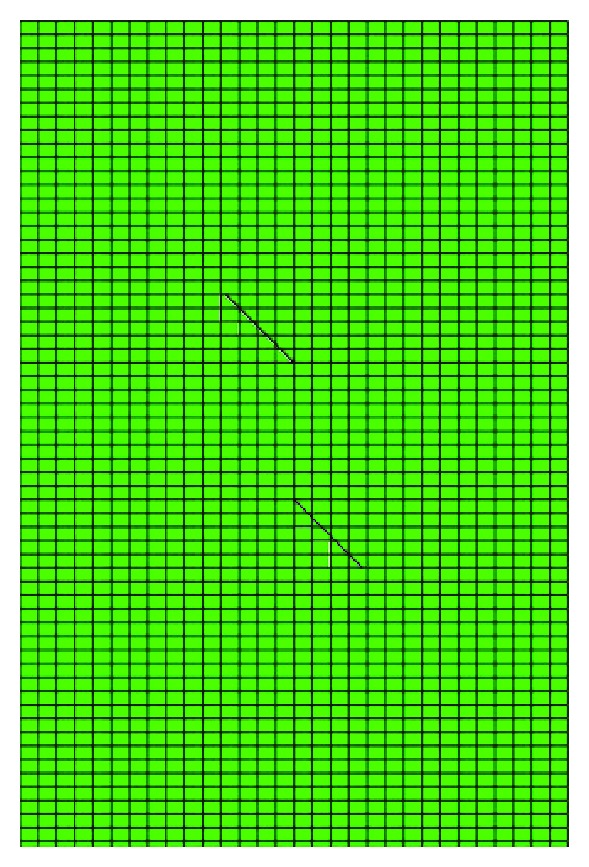
The numerical model of crack extension trajectory under uniaxial compression.

**Figure 8:**
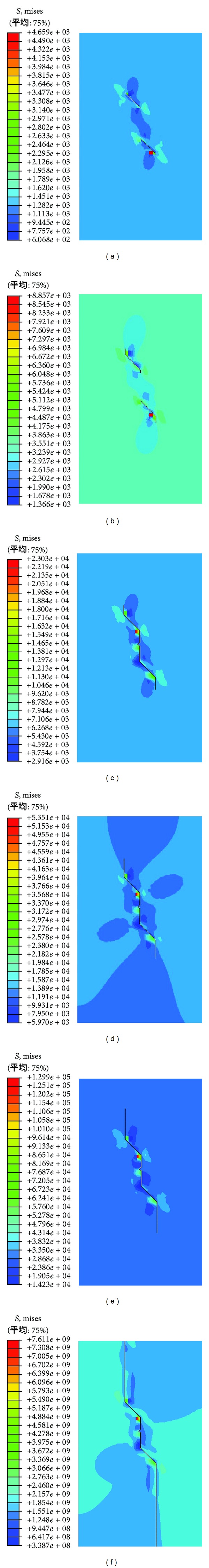
Crack propagated of rock sample under compression simulated by XFEM.

The simulations on the crack development are carried to examine the efficiency of XFEM, which is shown in Figures 7 and 8.

### 2.5. Simulations on Zonal Disintegration

The numerical tests are carried out on the rock samples containing hole with XFEM, and zonal disintegration is reproduced (Figure 10).

The maximum circumference tension stress criterion was taken as the initiate criterion.

The critical loading depends on the equation

(2) $$\begin{array}{c} K_{e}=\cos\frac{\theta }{2}\left[K_{I}\cos^{2}\left(\frac{\theta }{2}\right)-\frac{3}{2}K_{II}\sin\theta \right]\geq K_{IC}. \end{array}$$

That is, the fracture begins to develop when the equivalent stress intensity factor *K*_*e*_ is equal to or larger than the fracture toughness of the surrounding rocks *K*_*IC*_.

The angle of crack initiates is

(3) $$\begin{array}{c} \theta_{0}=\text{arccos}\frac{3K_{II}^{2}+\sqrt{K_{I}^{2}+8K_{I}^{2}K_{II}^{2}}}{K_{I}^{2}+9K_{II}^{2}}, \end{array}$$

where *K*_*II*_ = 0. Substitute it to (2) and (3), then:

(4) $$\begin{array}{c} K_{I}=K_{IC}. \end{array}$$

The stress intensity factor of circular fracture is [17]

(5) K1(r)=2P0πrsinθ(3−cos⁡θ) ×{(1+ra2r2)2ra(3cos⁡θ−1)3π2− (1−ra2r2)rsinθcos⁡θ2}.

Substitute (5) into (4). The criteria of zonal disintegration can be got by

(6) 2ra(3cos⁡θ−1)3π2σ1−rσ2sinθcos⁡θ2  ≥K1Crsinθ(3−cos⁡θ)2π.

The fracture angle is *θ*_0_ = 0, which means that the crack will initiate along the plane where it exists, where *K*_*e*_ is the equivalent stress intensity factor of fracture; *K*_*IC*_ is the fracture toughness; *θ*_0_ is the angle of crack initiates.

### 2.6. Simulation Procedure

**Figure 9:**
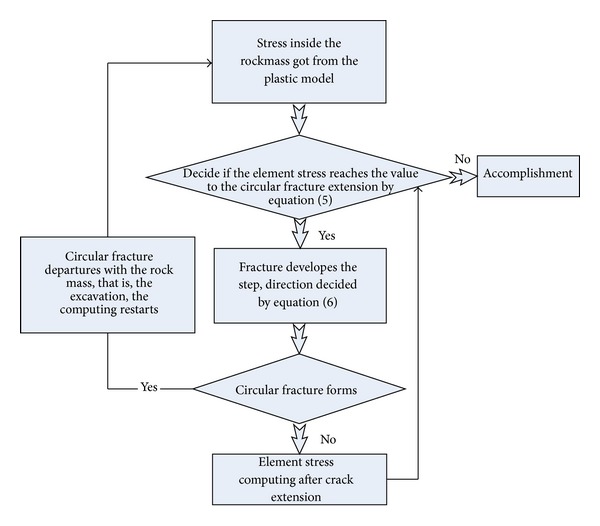
Block diagram of the simulating procedure of zonal disintegration.

The numerical process of zonal disintegration is shown in Figure 9.

### 2.7. Numerical Simulation of Zonal Disintegration of Deep Cavern

In order to research the forming mechanism deeply, the research on the damage procedure of zonal disintegration is carried on based on the geomechanical model test.

The diameter of model is 0.45 m, with the circle cavern of 160 mm in diameter, which is the same as the model test. The model is divided into 30000 elements (four-node). In order to simplify the issue, the hydraulic pressure is applied on the model, which is 0.5 MPa.

The numerical model are shown in Figure 8. The result and displacement surrounding cavern are in Figure 9.

**Figure 10:**
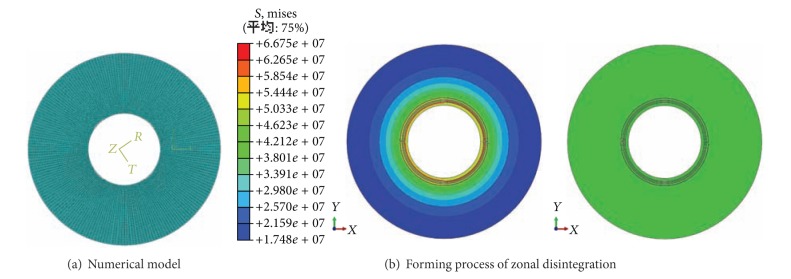
Zonal disintegration of circular model obtained with XFEM.

**Figure 11:**
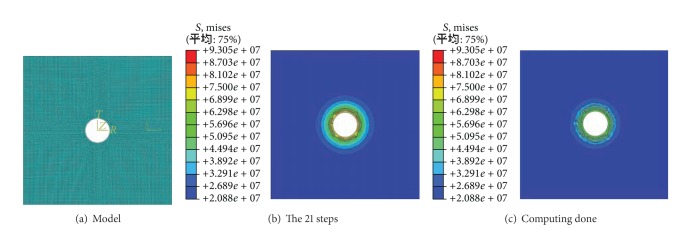
Zonal disintegration phenomenon in surrounding rock mass simulated with XFEM.

As Figures 10 and 11 show, there exist 4 circular fractures around the cavern. The result is similar to the model test. So the zonal disintegration is proved by using numerical simulation.

**Figure 12:**
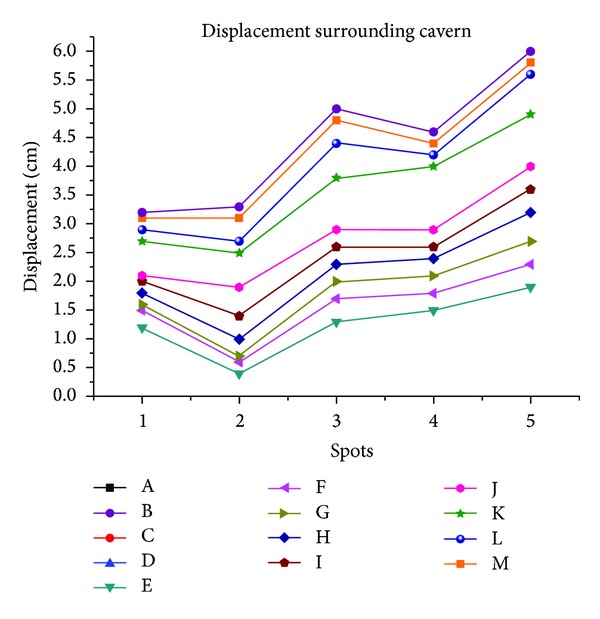
Displacement distribution surrounding the cavern.

And as Figure 12 shows, the deformation law is nonlinear like the result of model test.

### 2.8. Comparison between Model Test and Numerical Simulation

As shown in the photos of the fracture shapes of model test and numerical simulation, it can be found as follows.

Circular cracks propagated and coalesced to form a circular pattern. Four fracture zones were observed to surround the cavern, in accordance with the fractured shape obtained from the model test. Thus, the occurrence of zonal disintegration was confirmed in the model.

Comparing the fracture shape in numerical model (Figure 11) with that in the test (Figure 5), it is found that a system of cracks geometrically similar to the periphery of the tunnel characterised the zonal structure observed both in numerical simulation and the model. Based on the fracture shape of the model, the fracture radius was related to the cavern radius. Suppose that the radii of the fractured zone are *r*_*i*_  (*i* = 0,1, 2,…, *n*), and the radius of the cavern is *r*_0_. Thus, the radius of the fracture zones fulfills the following relation:

(7) $$\begin{array}{c} r_{i}=r_{0}\cdot \alpha^{i}. \end{array}$$

That is,

(8) $$\begin{array}{c} \frac{r_{1}}{r_{0}}=\frac{r_{2}}{r_{1}}=\cdots =\frac{r_{n}}{r_{n-1}}=\alpha ,\;\left(i=0,1,2,\ldots ,n\right). \end{array}$$

Some specialists believe that *α* is a constant value; that is, $\alpha =\sqrt{2}$ Oparin and Kurlenya [12]. However, after measuring the disassembled model, it was observed that *α* is not equal to $\sqrt{2}$; in actuality, *α* ≈ 1.2 in this paper.

### 2.9. Displacement Law in Surrounding Rocks

Figure 11 shows the displacement surrounding the cavern. The displacement in each measurement line presents an oscillating law in which the peak and valley values alternate with distance. This law is completely different from the law observed for shallow embedded tunnels, in which the displacement decreases monotonically with increasing distance from the tunnel wall. By comparing with the fracture shape of the model, it was observed that the region of peak values showing greater displacement is the fracture zone, whereas the region of minimum values showing less displacement is the intact zone. The additional displacement in the peak value area caused by the circular fracture increases the total displacement.

Comparing Figure 11 with Figure 5 reveals that the displacement law obtained from the numerical simulation is analogous to that obtained from model test. They both show the nonmonotonic law.

## 3. Conclusions

1. Zonal disintegration phenomenon is simulated by XFEM.
2. The criteria of crack propagation are got. And it is appropriate for the simulation of zonal disintegration.
3. The results between the geomechanics model test and numerical simulation are consistent. Both the two methods show that the fracture line is the concentric circles of the cave. There are 4 fractured zones that appear surrounding the cavern. And the radiuses of the fractured zones fulfill the geometric progression.

## Figures and Tables

**Table 1:** Physical-mechanical parameters of the prototype rock and model material.

Material type	Prototype	Similar material
Volume Weight/KN·m^−3^	2.62	2.62
Edef/Mpa	5250	175
Cohesion/Mpa	10	0.4
Friction angle	43	43
Uniaxial compressive strength/Mpa	29.08	1
Tensile strength/Mpa	10.22	0.674
Passion ratio	0.272	0.272
